# Relationship-Oriented Recovery System for Youth (RORSY): Clinical Protocol for Transition-Age Youth with Opioid Use Disorders

**DOI:** 10.24966/aad-7276/100144

**Published:** 2023-09-29

**Authors:** Aaron Hogue, Molly Bobek, Alexandra MacLean, Jeremiah A Schumm, Kevin Wenzel, Marc Fishman

**Affiliations:** 1 Partnership to End Addiction, New York, USA; 2 OneFifteen, Inc./Samaritan Behavioral Health, Inc., Wright State University, Dayton, USA; 3 Maryland Treatment Centers, Maryland, USA

**Keywords:** Adolescents, Family-based interventions, Opioid use disorder, Young adults

## Abstract

This article introduces the Relationship-Oriented Recovery System for Youth (RORSY) protocol, which is designed to increase uptake of Medications for Opioid Use Disorder (MOUD) and related services among adolescents and young adults. Youth exhibit alarmingly poor rates of MOUD initiation and adherence, OUD services involvement and long-term recovery success. RORSY attends to three developmentally unique recovery needs of this age group: assess and bolster youth recovery capital, prioritize involvement of concerned significant others, and use digital direct-to-consumer recovery supports. RORSY contains five evidence-informed intervention modules that can be flexibly tailored to meet the individual and relationship needs of a given youth: Relational Orientation, Youth Recovery Management Planning, Relational Recovery Management Planning, Relationship Skills Building, and Digital Recovery Support Planning. The article concludes with practice and policy recommendations for making relationship-building a top clinical priority for addressing youth OUD.

## Introduction

This article describes a set of modular clinical interventions designed to enhance treatment and recovery processes for adolescents and young adults with Opioid Use Disorder (OUD). It begins by discussing recent estimates of OUD prevalence among young persons aged 16 – 26 years, a group commonly called Transition-Age Youth (TAY). It then briefly introduces current best practices for OUD treatment and recovery interventions, focusing on the continuum of services for medications for Opioid Use Disorder (MOUD) and high-lighting the poor rates of MOUD initiation and adherence observed in this vulnerable group. It then describes three specific strategies for increasing the developmental attunement of MOUD services in order to meet the unique treatment and recovery needs of TAY. It culminates by articulating a new clinical protocol that interweaves these developmentally attuned strategies with existing evidence-based interventions for promoting OUD recovery among TAY: Relationship-Oriented Recovery System for Youth (RORSY). The five intervention modules of the RORSY protocol constitute an innovative approach to supporting and enhancing MOUD services for TAY: Relational Orientation, Youth Recovery Management Planning, Relational Recovery Management Planning, Relationship Skills Building and Digital Recovery Support Planning. The article concludes with practice and policy recommendations for making relationship-building a top clinical priority for addressing youth OUD.

## Opioid Use Disorder among Transition-Age Youth: Prevalence Rates, Treatment and Recovery Options, and Service Gaps

### Youth are the developmental epicenter of the nationwide opioid epidemic

The United States has experienced an opioid epidemic for nearly two decades, and rates of opioid misuse and opioid-related mortality climbed precipitously in 2020 [1]. Opioid misuse and related problems are especially alarming among TAY: Between 2002 and 2013 the rate of past-year heroin use more than doubled [2], and between 2006 and 2015 the rate of lethal opioid overdoses increased from 3.4 deaths to 5.3 deaths per 100,000 [3]. National data from 2019 [4] show nearly 1,800 youth initiate heroin or pain reliever misuse each day, and almost 300,000 meet criteria for OUD. Risk data indicate that 8–12% of those who engage in opioid misuse eventually develop OUD [5]. Because rates of opioid misuse and overdose are highest in this age range, TAY has been called the developmental epicenter of the epidemic. Although recent national surveys of high school students registered historic declines in substance use of all kinds, including prescription opioid misuse [6], levels remain alarmingly high despite this promising trend [7].

### MOUD services are available and effective for youth

MOUD, consisting of opioid agonist or antagonist medication combined with medication-supportive behavioral counseling, is the only evidence-based treatment for OUD [8]. MOUD is well-established for all age groups [9] and is recommended for TAY by national pediatric healthcare policy [10]. Initiation onto one of three FDA-approved MOUDs (buprenorphine, naltrexone and methadone) typically occurs during acute crisis-driven episodes of care (e.g., treatment of withdrawal or “detoxification”), after which enduring MOUD over time (“maintenance”) is a standard recommendation to prevent recurrence of opioid use problems (“relapse”). MOUD is often combined with ancillary behavioral and other recovery supports intended to support opioid abstinence and address other Substance Use Disorders (SUDs) and co-occurring mental health problems [9].

**Figure 1:**
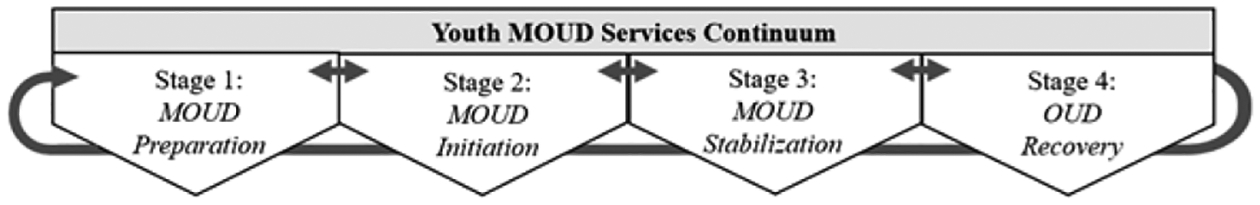
Youth MOUD services continuum.

As depicted in figure 1, MOUD services can be conceptualized as a continuum, sometimes called a services cascade, consisting of the typical sequence of intervention activities experienced by any given youth as they progress through the MOUD service system. The MOUD services continuum is anchored by four overlapping stages [11]. Stage 1: MOUD Preparation includes identification, referral, and enrollment of youth in MOUD services, including re-enrollment following recovery lapses. Stage 2: MOUD Initiation includes initial evaluation and medication induction. Stage 3: MOUD Stabilization includes dose titration and early response, which is often unstable and may include withdrawal management. Stage 4: OUD Recovery includes stability monitoring, relapse prevention, and improvement in overall health and quality of life. Importantly, behavioral interventions for substance use and co-occurring disorders are often integrated throughout Stages 2–4 of the MOUD cascade. Note that youth who enter MOUD services typically experience episodic increases and decreases in opioid use-that is, a chronic course-of-disorder marked by regular use, remission, and recurrence-over a given time span [12]. For this reason, movement along the continuum is rarely linear, in that many youth transition both forward and backward (i.e., re-entering earlier in the continuum following a recurrence of problems) across stages.

Focusing on Stage 4: OUD Recovery, Recovery Support Services (RSS) for OUD comprise a range of interventions to promote sustained efforts to eschew or reduce OUD and improve wellness. Over the last decade-plus, shifts in policy and insurance practices have vastly expanded the availability, accessibility, and diversity of RSS [13], making RSS a mainstay of the MOUD services continuum. RSS can be organized into three broad categories: (1) Professional RSS: services offered by licensed clinicians in the context of a provider-client relationship. Professional RSS are typically adjuncts to or extensions of Stage 3: MOUD Initiation interventions, as in continuing care models, as part of an ongoing monitoring and maintenance phase of treatment. (2) Peer/Community RSS: support offered by persons who have similar lived experiences in the context of a peer-to-peer relationship. These include peer recovery coaching, sober educational settings, recovery community centers, and mutual help groups that usually combine peer support via shared recovery experiences during group meetings and mentoring relationships with senior peers (aka sponsors) outside meetings. (3) Direct-to-Consumer (DTC) RSS: supports offered by social media or other information brokers that are accessed directly by affected persons. These include standardized (e.g., self-help books, website bulletins) and tailored (e.g., phone or digital helplines) educational and motivational materials. When individuals use DTC RSS without intercession from an external agent, this is considered an “unassisted” pathway to recovery [14]. It bears emphasizing that the RSS marketplace in all three categories is dominated by services aimed at individual youth directly rather than at families [15].

### Youth with OUD have poor rates of MOUD initiation, adherence and duration

The national OUD system of care is moving rapidly to increase MOUD availability for TAY [10]. Despite these efforts, only a fraction of youth with OUD receives any treatment, and even fewer receive MOUD [16], even after an opioid overdose episode [17]. Studies of MOUD services report universally low enrollment rates for TAY, in the area of 10–35% among those in need [17–19]. One study [20] reviewed Medicaid claims across service settings in 11 states and found that only 24% of youth with OUD received medication; half received behavioral services without MOUD; and there were marked disparities in MOUD access disfavoring younger, female, African American, and/or Latinx youth. Moreover, TAY who do initiate MOUD are significantly less likely to remain medication adherent compared to adults [21,22]. Even youth who complete episodes of residential OUD treatment show low MOUD initiation and post-residence continuation rates [23,24]. It is especially difficult to support TAY in remaining on MOUD across the months-to-years needed to accrue stable benefits. One-fourth who initiate MOUD leave treatment after one week, and most studies place one-year adherence rates between 9–17% [25,26]. Low medication adherence during the OUD recovery phase is highly problematic due to the strong association between MOUD duration and positive outcomes among TAY [27]. Altogether, these alarmingly poor rates of youth MOUD adherence and duration (collectively, MOUD “uptake”) critically undermine national efforts to curb the youth opioid crisis and support effective recovery.

## Promoting a Developmental Approach to MOUD Services among TAY

### Taking stock of developmental science to enhance MOUD uptake among youth

To address the OUD crisis among TAY, effective remedies for well-documented barriers to MOUD uptake are urgently needed. Developmental challenges navigated by TAY that specifically impact SUD services uptake include feelings of invincibility, decreased salience of SU consequences, aversion to or inconsistency in accessing healthcare, rapid fluctuations in motivation to attend counseling, and variable effectiveness of family leverage to motivate attendance, including pushback against parental dependence [28–30]. Prevailing theories about developmentally tailored approaches to engaging TAY in SU treatment are invariably grounded in Arnett’s framework for emerging adulthood [31,32]. Arnett’s work synthesizes existing developmental science for the 16–26 age range to identify discrete developmental challenges that pervade the beliefs and behaviors of TAY, including how those challenges intersect with SU issues [29,30,33,34]. Using Arnett’s framework, SU services engagement factors for TAY can be specified along three focal dimensions: (a) SU Risk and Protection Indicators such as peer SU consumption and attitudes, housing stability, family and peer/community supports, stressful events, national and community SU attitudes and norms, self-regulation capacity, and co-occurring behavioral disorders; (b) Service Enrollment Barriers such as low conscientiousness, extrinsic versus intrinsic motivation, low treatment stigma, and severity of SU problems as well as low awareness of problems; and (c) Independence Factors such as education/work aspirations, social capital, family involvement, financial and insurance support, self-sufficiency as well as subjective sense of self-sufficiency, which may be exaggerated.

Independence factors in particular exert a heavy influence on MOUD uptake in TAY. “Independence” is defined as the degree to which a youth is self-sufficient within their intersecting social networks. To help erode multifaceted MOUD uptake barriers experienced by youth, RSS should be developmentally calibrated to directly address the independence factors that drive youth MOUD engagement. While there is frequent tension between actual levels of emerging or partial independence and a developmentally normative, exaggerated perception of full independence with pushback against assistance (“Don’t tell me what to do…”), it is usually helpful to appeal to aspirational self-efficacy. Examples of independence-related developmental themes that can be clinically leveraged in this fashion include: conceptualize OUD as a chronic medical illness that a young person takes control over with ongoing recovery tools; acknowledge the normalcy of autonomy-seeking (including self-determination of health-related decisions) and how this is a healthy maturational trope; emphasize informed decision-making and collaboration about MOUD rather than rote compliance; and strengthen social networks that can support MOUD goals [28,34]. In this vein, below are described three specific ways in which MOUD services can be developmentally attuned to help erode barriers to MOUD uptake among TAY.

### Developmental calibration #1: systematically assess and bolster youth recovery capital

Comprehensive models of youth SUD “recovery capital” [35,36] stipulate four basic domains of instrumental resources for supporting SUD recovery: financial resources that enable access to recovery supports and buffer youth from life stressors; individual resources (e.g., cognitive functioning, temperament) used to achieve personal goals; social resources generated through relationships with others; and community resources that include recovery supports and community attitudes. As the starting point for developmental calibration of youth MOUD services, clinical assessment procedures should identify the unique recovery capital profile of a given youth, focusing on independence factors such as education and work aspirations, routine involvement of Concerned Significant Others (CSO), and plans for independent living. Assessment of such factors lays the groundwork for formulating client-tailored interventions that aim to bolster recovery resources in the most salient capital domains, which can also engender synergistic interactions among domains [36]. Even within the relatively narrow TAY age range, assessment must account for developmental variation in the interaction between youth independence and expression of SUD risk and protective factors. For example, as autonomy in decision-making increases from mid-adolescence to near-adulthood, family involvement viewed by youth as supportive and/or collaborative is much more influential on motivation for SUD treatment than involvement viewed as coercive [37].

### Developmental calibration #2: prioritize involvement of concerned significant others

Although family resources are a fundamental domain of youth recovery capital, CSO-focused strategies to support MOUD uptake are frustratingly scarce. CSO (defined as family of origin, romantic partners, and/or family-of-choice members) represent primary risk and protective factors and contexts of developmental influence for youth SUD, and typically CSO remain highly involved (including financially) with substance-using youth [38]. Multiple reviews by our team and others report that empirical support for CSO-focused treatments is among the strongest for SUD treatment engagement and outcomes among both adolescents [39] and adults [40]. CSO-focused interventions can also improve coping skills and quality-of-life indicators among affected family members [41]. Also, parent-to-parent programs, which connect parents of children with health disorders to “veteran” parents who have faced similar issues, have shown efficacy in improving family coping skills and help-seeking [42]. Yet despite this wealth of evidence, CSO are rarely incorporated systematically in treatment and recovery activities for youth SUD in general or for youth OUD specifically [43]. A core principle is that for almost all other health concerns it is considered routine, even an obligation, to help a loved one (especially a family member) who faces challenges and may be having difficulty with optimizing effective utilization of treatment services. But this is not so in SUD treatment, for many reasons. To be sure, prominent barriers against involving CSO in SUD/OUD services exist among both providers (e.g., biases against CSO as causes of SUD/OUD problems, lack of skills or motivation to pursue CSO outreach, beliefs that youth with SUD/OUD need unilateral individuation from parents [43,44]), beliefs that only internal insight and motivation can produce behavior change, and among CSO themselves (e.g., demoralization about proving support, reticence to engage with SUD/OUD care [45]). Even so, CSO involvement appears to be an enormous asset for sustaining long-term SUD/OUD recovery among TAY [44,46].

### Developmental calibration #3: use digital direct-to-consumer recovery supports

Given the near-ubiquity of smartphones and widespread use of the internet among TAY [47], MOUD service providers have unprecedented opportunities to employ a comprehensive range of digital DTC interventions for youth. TAY are developmentally primed for engaging in digital recovery supports such as automated text messaging, self-directed internet-based courses and internet support via social media platforms. This includes peer-to-peer coaching services, peer networking forums, and online support groups (often moderated by professionals) that allow TAY to seek support from persons with similar lived experience [48]. A key caveat is that research evaluating the effectiveness of digital DTC RSS for youth is scarce, and there are several youth engagement barriers to surmount, including disappointing rates of intervention completion, concerns about privacy and security, and design quality and packaging features that fit uneasily with user expectations [35,48].

## Filling OUD Service Gaps for TAY: Relationship-Oriented Recovery System for Youth

### Developmental and clinical foundations for RORSY

This section details a clinical protocol specifically designed to increase MOUD uptake among TAY: Relationship-Oriented Recovery System for Youth (RORSY). RORSY is anchored in the three research-grounded development calibration principles outlined in the previous section: bolster youth recovery capital, prioritize CSO involvement in OUD services, and utilize digital recovery supports. As such, it contains three main innovations designed to address existing gaps in youth MOUD initiation, adherence and duration. First, it brings the arsenal of relationship-oriented interventions for SUD to bear on youth OUD specifically. Despite their exceptional research portfolio, CSO-focused models for SUD have not been widely adopted in everyday care, primarily because they are costly and cumbersome to implement due to multicomponent training and quality procedures [49]. However, recent breakthroughs in distilling the core techniques of CSO-focused models for SUD [50–52] make these techniques clinically accessible, flexible for client-centered intervention tailoring, and pragmatic in a variety of treatment settings [53]. Unlike more formal family therapies that are often regarded as alternatives to usual care, the RORSY protocol as articulated here is intended to have impact in informing an approach to care that can be adapted and integrated into usual care, without requiring rigid implementation with high fidelity [49]. Second, RORSY intervention components are tailored to meet the individual and relationship needs of a given youth. The launch point for RORSY is clinical assessment of youth recovery capital to assess relationship needs, independent living and coping skills deficits, whether CSO are tenable candidates for involvement in MOUD services, and whether and how CSO can support the youth’s self-sufficiency goals. Assessment-driven personalized interventions of this kind hold great promise for optimizing intervention effectiveness among groups with diverse symptom profiles [54]. Third, RORSY features CSO-oriented recovery management planning procedures to assess MOUD adherence status, reinforce youth progress toward abstinence and recovery, plan how to react in the event of overdose, and link youth to online digital RSS. CSO-oriented recovery management also includes connecting CSO to their own digital RSS that are designed to fortify theirself-care and their ability to connect to and support their OUD-affected loved one, which can tangibly promote the loved one’s recovery [46,55].

**Figure 2:**
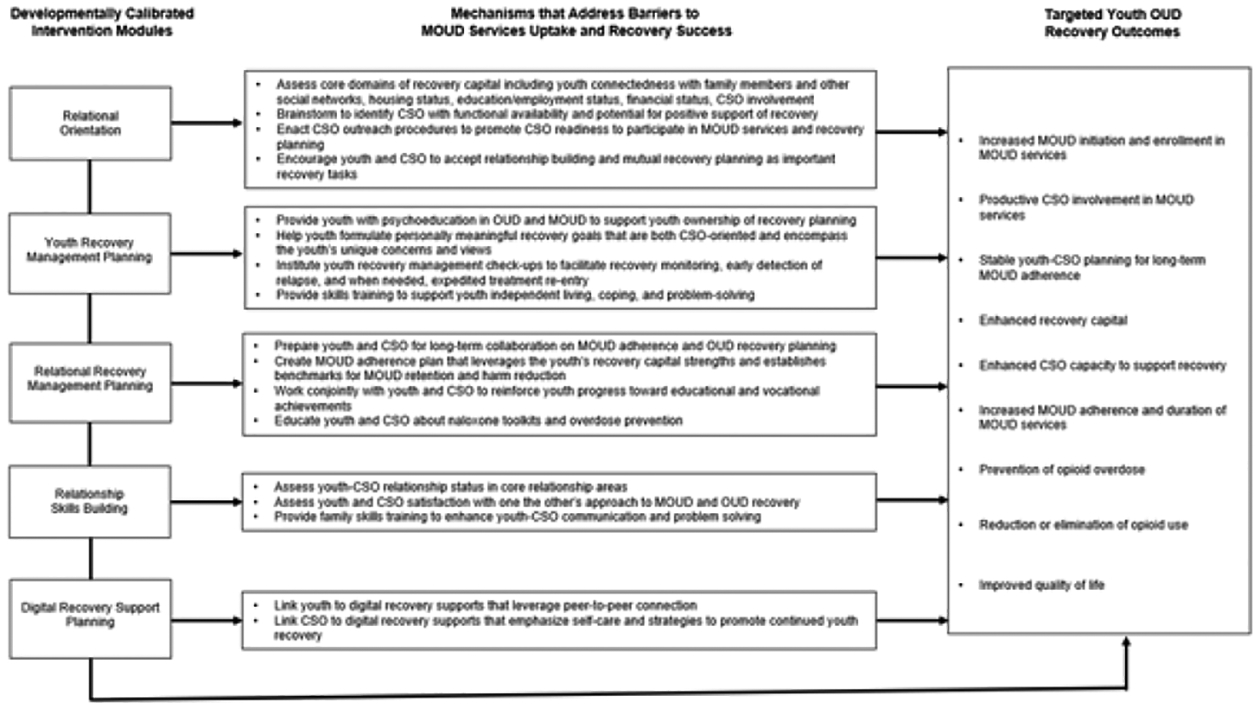
RORSY intervention modules, mechanisms and targeted outcomes.

Figure 2 displays RORSY’s five intervention components, primary intervention mechanisms to address barriers to MOUD services uptake and recovery success, and targeted youth OUD recovery outcomes. As detailed below, these five modular components are meant to be delivered to whatever extent is clinically indicated based on case status and client goals [53]. Module 1: Relational Orientation, which is delivered first for every case, prescribes a standard order of four intervention Tasks (“standard”). The other four modules and their various submodule interventions are delivered as indicated (“optional”). Modules can be completed in one session, staggered across sessions, and/or interspersed with other individual or CSO-focused interventions. Time needed to complete each module varies based on the profile of the given youth and CSO, practice habits of the given clinician and case progress. To account for the wide diversity in CSO configuration experienced by TAY, during Module 1 clinicians assess youth independence status to judge whether and which CSO are promising candidates for involvement in MOUD services, and how invited CSO can serve as sources of recovery support for youth self-sufficiency goals. To be sure, many TAY with OUD have minimal support networks, and sometimes negligible involvement with CSO of any kind. In such cases RORSY modules can be delivered with youth alone, which itself advances a transformative clinical perspective: Even when working individually with a youth client, it is highly valuable for these youth to adopt relationship-oriented OUD recovery habits and options.

### RORSY module 1: relational orientation

#### Task 1: Youth independence/interdependence assessment:

Per Arnett’s framework, an overarching developmental theme for TAY is independence status [31,32]. Because youth in(ter)dependence factors, including the degree and salubrity of youth connectedness with family/social networks, are pervasive in all aspects of TAY functioning, they must be integrated when tailoring strategies to promote MOUD uptake. Accordingly, clinicians assess housing status (e.g., living with caregivers/partners, living independently, institutionalized), education/employment status, financial status, and CSO involvement.

#### Task 2: Youth nomination of CSO:

Based on results of Task 1, clinicians help the youth identify CSO with potential for functional availability and positive support of recovery. Central facets of youth in(ter)dependence then shape how clinicians engage a given youth in MOUD services with regard to addressing stigma and perceived treatment value within their social networks, deciding whether and which CSO to involve in MOUD services, and strengthening a social safety net to support MOUD uptake [28]. For all youth, it is important to explore how youth find and define family potentially beyond biological or legal ties, and to honor this broader “family of choice” in the CSO nomination process. It may be very important to consider inviting more than one CSO to participate in order to create balance in perspectives, provide additional support for nominated family members, and/or build a support network as broad as possible. When a supportive CSO is not currently available, and/or a youth is reluctant to nominate potential CSO, clinicians can deliver youth-only elements of the remaining RORSY modules, while also periodically revisiting whether or which CSO may be available to invite. One strategy to accomplish this task is to encourage youth to brainstorm and list everybody they can think of, and then start a collaborative process of weighing options, thinking through the specifics of how each person might help them, and so forth.

#### Task 3: CSO engagement:

To make initial connections with nominated CSO, clinicians enact outreach procedures to address potential logistic and attitudinal barriers and enhance CSO readiness and motivation to participate. This includes anticipating how family resources and dynamics could impact participation [56], building a therapeutic alliance with CSO [57], and providing rationale for participation that accounts for both youth- and CSO-specific concerns [58]. Clinicians should consider a stepwise “foot-in-the-door” approach to engaging CSO for an initial session that involves treatment planning and exploration of options for how to support youth in their recovery. When CSO participate in a first session, clinicians promote long-term CSO engagement in OUD services by instilling hope and involving them in recovery goals. Clinicians join with CSO by showing respect, curiosity, and acceptance; expressing appreciation and empathy regarding past frustrations over the youth’s condition and behavior; using relevant self-disclosure to establish connection; and promoting participation by validating topics and concerns they raise. Clinicians then formulate MOUD service goals in a manner that involves CSO in a meaningful and pragmatic way to support MOUD services [59]. When initial outreach to CSO proves especially challenging, clinicians can employ principles of strategic structural systems engagement [57] to recognize treatment-incompatible agendas of CSO, and how these reduce the likelihood of CSO participation; identify who can act as a reliable family messenger, and who has power to influence other members to potentially attend; and provide rationale for treatment that accounts for the specific concerns of CSO and perhaps other key family members [58]. As clinicians work to engage CSO, it is imperative they explore the influences of race, ethnicity, and culture on the engagement process [60].

#### Task 4: OUD relational reframe:

Clinicians use relational reframing techniques to shift the focus of OUD services from exclusively fixing youth symptoms to improving the quality of youth-CSO relations, which will then strengthen the youth’s OUD recovery network. This occurs when clinicians ask patients to shift attention from an exclusive focus on individual behaviors to a broader view of interpersonal relationships. This typically begins by encouraging youth and CSO to accept relationship building and mutual goal-setting as an important recovery task [51]. Clinicians assert that acknowledging, understanding, and repairing relationship problems can be an effective way to address individual problems and, for youth with OUD, bolster MOUD recovery support. Basic approaches to delivering a relational reframe include: identifying sequences of behaviors or emotions involving CSO that precede, or directly cause, an OUD-related problem; focusing directly on the impact an OUD-related problem has on the actions, thoughts, and feelings of both youth and CSO; and championing relationship repair or improvement [61,62].

### RORSY module 2: youth recovery management planning

#### Youth leadership in MOUD adherence planning:

Clinicians engage with youth and CSO to support youth autonomy, while also helping youth understand the limitations of assertions of autonomy that may have led to problematic decisions in the past, and to nourish developmentally appropriate youth leadership on their own healthcare needs, with regard to MOUD services. This includes attending to key youth independence factors related to OUD recovery (e.g., conceptualize OUD as a chronic condition, explore and support incremental non-abstinent SU goals, address wider social network change). Youth leadership begins with helping the given youth formulate personally meaningful recovery goals that are both CSO-oriented and encompass the youth’s unique concerns and views [63]. As youth become more sophisticated in their leadership, they can also learn to accept the limits of their judgement when they are under the influence and/or otherwise in the throes of OUD. Clinicians guide youth in communicating more effectively with CSO to voice their fears, frustrations and needs. Relatedly, clinicians help youth gain incremental authority of educational facts related to OUD and MOUD, including OUD norms and prevalence rates; medication formulations and their respective dosing procedures; medication benefits, expected course, and potential side effects; and factors related to MOUD stigma [64]. To facilitate MOUD education, clinicians utilize an archive of colorful infographics to facilitate discussion of issues impacting youth and CSO participation in MOUD services.

#### Youth recovery management check-up:

Recovery Management Check-ups (RMCs) are proactive, regularly scheduled SUD recovery checkups that provide (a) routine assessments and personalized feedback for youth on the status of their OUD recovery and risks and (b) motivation-based service linkage and retention protocols to help youth secure recovery supports over extended periods [65]. Although originally conceived as a case management procedure, the goals of RMCs translate well into the more commonly understood use of the term “check-up” as implemented by a longitudinal care team. RCMs are instituted as Stage 4: OUD Recovery commences, with clinicians facilitating recovery monitoring, early detection of relapse, and when needed, expedited treatment re-entry. RMCs focus on managing addiction as a chronic condition, attending to SUD service barriers and strategies to access care, and enhancing youth motivation for treatment. RMCs are backed by cost-effectiveness data [66], have been implemented with strong effect sizes for adults with OUD [67] and for adolescents with SUD [68], and are readily mounted and managed on digital platforms [65].

#### Youth independence skills and coping exercises:

Based on the Task 1 youth independence assessment, clinicians can selectively support youth in acquiring competencies for the transition to independent living-e.g., housing, financial management, education, and employment needs-by using standardized skills training protocols. These elements are likely more appealing to youth than discussions about treatment implementation (e.g., appointments, medication adherence), and can be linked contingently as the natural return on the investment in those more treatment-focused mechanics. In addition, coping-focused interventions can help youth recognize and modulate impulsivity, depressed and anxious moods, and stress reactivity. This includes treatment of co-occurring disorders and addressing affective dysregulation, for example: appropriate psychiatric treatment, anger management exercises to reduce or interrupt expressions of anger/aggression that yield maladaptive outcomes [69]; and relaxation exercises to reduce arousal levels that render youth susceptible to compromised reasoning and decision-making, difficulty with concentration, sleep problems, and reliance on SU to moderate stress [70]. These various exercises, as with all skills training interventions contained in the RORSY protocol, are meant to be practiced in session wherein clinicians can provide client-tailored coaching and feedback. A standard skills training framework is: (1) explain the rationale; (2) model the skill; and (3) have the youth practice the skill with provider coaching.

### RORSY module 3: relational recovery management planning

#### Youth & CSO collaborative MOUD adherence planning:

Clinicians prepare youth and CSO for long-term MOUD adherence and OUD recovery planning. This includes emphasizing the central role of social network involvement in MOUD maintenance and the OUD recovery process; anticipating how current social capital resources impact MOUD adherence; and dismantling MOUD-related stigma [44,71,72]. Clinicians also draw from (a) motivational strategies to address ambivalence and stages of change [73] and (b) family and couple therapy strategies on engineering productive youth-CSO dialogue for youth with SUD that help contextualize MOUD adherence within the youth’s network of supports [59,63]. This concludes with specifying a collaboratively drawn MOUD Adherence Plan that reflects the youth’s OUD profile, aims to leverage recovery capital strengths and supports, and establishes benchmarks for MOUD retention and harm reduction [74]. The Adherence Plan involves collaborative monitoring of the youth’s MOUD by identifying MOUD compliance goals, tracking success in meeting these goals, and CSO verbally reinforcing this success.

#### Youth & CSO education and vocation planning:

Utilizing principles of assertive continuing care for youth SUD, clinicians work conjointly with youth and CSO to reinforce youth progress toward quality-of-life benchmarks. Central among these are educational and vocational achievements. Youth with OUD typically have social lives anchored in SU-related behavior, making it a challenge to pursue and achieve developmental milestones in education and vocation planning. Clinicians employ case management [75] and goal-setting interventions [76] to address these deficits; and per a collaborative decision-making approach, CSO can support and be involved in numerous aspects of these activities.

#### CSO-involved overdose prevention education:

Clinicians deliver standardized layperson-targeted interventions to educate youth and CSO about naloxone toolkits and overdose prevention [77]. This includes corrective information related to myths of naloxone availability (e.g., having access to naloxone will enable and increase likelihood that youth will relapse). Clinicians also emphasize that the best overdose prevention is persistent adherence to MOUD. Clinicians work to achieve consensus between youth and CSO on network-wide safety and overdose prevention plans that are suited to current and planned youth living arrangements. Youth and CSO are meant to practice overdose training in session under the hands-on guidance of clinicians.

### RORSY module 4: relationship skills building

#### Youth-CSO relationship assessment:

Clinicians use an assessment tool derived from CRAFT [78], which is effective among youth [79] and adults [80]. A brief scale completed separately by youth and CSO identifies satisfaction with the youth-CSO relationship(s) in eight areas: household duties, social activities, allowance/money management, communication, relationship affection, school/job, emotional support, independence. This tool also assesses satisfaction with the other’s attitudes and approach to MOUD and to OUD recovery. Also, clinicians assess and monitor indicators of partner violence between youth and CSO that would mitigate or negate the possibility of relationship skills building.

#### Youth-CSO communication and problem solving:

Clinicians use family skills training techniques that are borrowed from Family Behavior Therapy (FBT; [81]) to enhance youth-CSO communication and problem solving. FBT has demonstrated positive outcomes in substance-using adolescent [82], young adult [83] and mature adult [84] populations. Skills building exercises include reciprocity awareness (expressed appreciation for past actions combined with reassurance that acknowledged behaviors will be repeated), positive request (asking effectively for what one wants and responding more effectively to requests from others), family communication (e.g., “I” statements, active listening, taking partial responsibility), and family decision-making and problem-solving (e.g., defining problems, delineating preferred outcomes, monitoring and evaluating solutions).

### RORSY module 5: digital recovery support planning

Clinicians link both youth and CSO to free, Digital Recovery Support Services (D-RSS) designed to support youth with SUD, including OUD, and their families. For youth, a variety of D-RSS that leverage peer-to-peer connection-via video meetings, message boards, and online groups accessible by phone apps or websites-are thought to bolster OUD recovery by offering 24-hour social support, connection to recovery-supportive persons who reinforce healthy behavior and accountability, and exposure to coping and well-being strategies [48,85,86]. CSO can be linked to a comprehensive suite of D-RSS sponsored by Partnership to End Addiction (www.drugfree.org): helpline (one-on-one sessions with specialists trained in supportive counseling techniques); family peer coaching (parent-to-parent support from peer coaches with their own history of parenting a youth with SU problems, which may improve CSO use of positive communication and behavioral strategies [55]); mobile messaging (personalized automated daily text messaging that offers advice for improved CSO-youth communication, links to SUD education resources, and directions for accessing additional supports); peer support communities (online peer-facilitated groups that provide both support and skill development); and self-directed e-learning curricula to help CSO learn effective coping and caregiving strategies. These tools need further empirical validation but hold great promise because of their user-friendly, family-empowering qualities, with easy access and scalability.

For both youth and CSO, actively linking to D-RSS is conceptualized as a two-part process: (1) Use motivational principles [73] to enhance readiness to engage in support options. For CSO, clinicians also emphasize potential benefits of improved self-care for boosting CSO capacity to support youth engagement in OUD services and augmenting the youth’s overall recovery capital. (2) Enact client-centered principles of “warm” service referral [87] that emphasize collaboration in selecting services and guide clinicians to elicit client input during the D-RSS search and trial process. RORSY employs recorded virtual tours of available D-RSS and sign-up procedures that clinicians and youth/CSO can jointly view, discuss, and activate.

## Making Relationships a Priority for Youth with OUD: Practice and Policy Pathways

### Helping clinicians be relationship oriented: following a path toward workforce training

As described above, CSO-focused interventions have strong empirical support in promoting treatment engagement and positive outcomes for youth with SU problems. RORSY contains several of the intervention techniques that have been identified as core elements of family therapy for youth SUD [51,88] and have been directly linked to long-term clinical gains [89,90]: relational orientation, relational reframing, youth and CSO treatment engagement, and family interaction enhancement. Certainly, RORSY is not unique in emphasizing a CSO-focused approach to youth SU problems. Regarding SUD generally, another CSO-focused model shown to boost treatment engagement is Community Reinforcement and Family Training (CRAFT; [78]). A main component of CRAFT for TAY is treatment entry training, which focuses on training CSO to recognize appropriate times for them to suggest treatment, employ effective motivational strategies to endorse entry, and have treatment options available at the time a decision is made to enter [91]. CRAFT has been shown to promote enrollment in SUD services, with more intensive family training producing better enrollment rates [79,80].

Regarding OUD specifically, one promising innovation is the Youth Opioid Recovery Support (YORS) intervention [21,92], a multi-component protocol to enhance MOUD adherence and decrease opioid relapse. YORS actively incorporates CSO in multiple facets of youth MOUD services via role induction, MOUD education, and collaborative treatment planning that includes CSO-involved contingencies for various course-of-care scenarios that might occur during recovery. When youth drop out of MOUD services, YORS increases CSO activation via phone calls, text messaging, and conjoint treatment sessions that leverage CSO-youth relations to bolster recovery success. A case series showed YORS benefits for increasing engagement and retention in MOUD services [75]. Two small controlled studies found that compared to usual care, youth in YORS received more medication doses, had lower relapse rates and had longer time to relapse [23,93].

Yet, in order for relationship-focused protocols like RORSY and others to be viable options within the youth MOUD service system, there need to be procedures for training the clinical workforce to deliver relationship-focused intervention techniques. One pathway to workforce training is enlisting manualized family therapy models for SUD that feature robust quality assurance procedures anchored by multicomponent training toolkits, guidelines for ongoing training and consultation from model experts, and implementation supports and fidelity tracking methods that feed therapy session data back to providers [40]. Whereas such procedures reliably boost protocol fidelity, they also incur substantial financial and resource costs for hiring model purveyors, conducting initial training and maintaining ongoing certification [49]. In addition, manualized family therapies prescribe numerous complex treatment procedures, often with a fixed intervention sequence-features that can inhibit the client-centered treatment selection and tailoring practices favored by community clinicians [53].

A second pathway is training clinicians in core elements of relationship-oriented interventions utilizing training methods that maximize practicality and cost-efficiency [94]. This pathway has some key advantages including greater ease of adoption, scalability, amenability to customized adaptation, and the ability to “layer” family approaches onto other usual care interventions, rather than viewing it as a wholesale replacement for usual care. The RORSY protocol is intended to provide one such roadmap for this more flexible approach. Though still in developmental stages, initial efforts to develop online training procedures in core relationship-oriented treatment techniques for youth SUD have produced gains in clinician reliability and accuracy when coding video vignettes for technique use [95,96] and when self-reporting on delivery of relationship-oriented techniques with their own cases [97]. Overall, online learning management systems for training community clinicians in the full spectrum of evidence-based behavioral interventions are advancing in waves, though it remains to be seen when, how, and for whom such methods meaningfully augment clinician performance and client outcomes [98].

### Helping clinicians be family flexible: mapping multiple paths of family involvement

Involving CSO in services for youth OUD is extremely challenging. The barriers to CSO involvement mentioned above are indeed pervasive and formidable. On the family side: CSO who might be supportive of youth treatment planning are often difficult to reach; ambivalent about participating in OUD services or in medical services of any kind; stretched thin by their own challenges and their responsibilities to other family members; and/or unconvinced that they can productively contribute to the youth’s recovery. On the youth side: TAY are often themselves difficult to engage in services, and when they do, participate sporadically; manifest poor medication adherence, consistent relapse problems, and severe OUD-related behavioral and medical health issues that consume treatment planning; and question or oppose CSO involvement of any kind. Faced with such barriers, often piling one upon another, it can seem daunting or even impossible to make CSO involvement a treatment priority, or even a realistic goal.

The RORSY protocol contains a thick roster of interventions designed to help clinicians address such barriers and ultimately increase CSO involvement in youth OUD recovery planning. But it bears repeating emphasis on the protocol’s modularity and flexibility: Clinicians are encouraged to choose which aspects of the protocol might be useful for which cases, and to what degree. That is, the protocol is designed to honor the clinical reality that there are multiple paths of CSO involvement available for any given clinician and their given TAY patient. One goal may be to convene a single meeting with CSO, in order to educate them broadly about their youth’s OUD recovery plan, and perhaps gain their expressed approval. A different goal may be to convene a few meetings with CSO in order to collaboratively articulate challenges to the youth’s recovery plan, and perhaps gain their expressed intentions to be actively supportive. An even more ambitious goal is involving CSO as integral participants in OUD services and committed partners in the youth’s recovery process. Various tasks and modules of the RORSY protocol can guide clinicians in traversing any number of family involvement paths. As such, RORSY does not present clinicians with an all-or-nothing scenario of standardized protocol delivery (i.e., drink from the firehose), but a flexible menu of manageable interventions for selective treatment planning (i.e., move the needle).

### Helping treatment providers become family committed: forging a path toward organizational change

While evidence for the effectiveness of CSO involvement in youth SUD care continues to grow, examinations of the current state of treatment services nationally indicate that agencies and treatment planning policies do not prioritize family-focused outreach or family-based services [99]. This status quo must be changed. Research with child and family behavioral health agencies shows that amendments in organizational policies and practices concerning family participation can directly impact family member attendance and increase delivery of family-based services [100]. We are advocating for a foundational shift whereby youth-serving behavioral health organizations become family-committed: Steadfastly determined to pursue the crucial goal of active family involvement in services for all youth who enter care. Comprehensive roadmaps of evidence-based practices for involving CSO in SUD treatment and recovery already exist [99]. Discovering how to put those practices to work for MOUD services in particular and SUD services writ large-that is, how to achieve adoption and implementation successes with clinicians, healthcare organizations, regulatory agencies, and of course families themselves-is the challenge before us.
